# Health-Related Quality of Life After Laparoscopic Cholecystectomy

**DOI:** 10.7759/cureus.26739

**Published:** 2022-07-11

**Authors:** Qurrat Al Ain Atif, Mumtaz A Khan, Faisal Nadeem, Muneeb Ullah

**Affiliations:** 1 Surgery, Darent Valley Hospital, Dartford, GBR; 2 General Surgery, Pakistan Institute of Medical Sciences, Islamabad, PAK; 3 Laparoscopic Surgery, Maroof International Hospital, Islamabad, PAK; 4 General Surgery, Maroof International Hospital, Islamabad, PAK

**Keywords:** gallstone disease, laparoscopic cholecystectomy, cholelithiasis, gastrointestinal quality of life indicator (giqli), health-related quality of life (hrqol)

## Abstract

Background

This study aimed to determine the mean improvement in the quality of life (QoL) after laparoscopic cholecystectomy (LC) in patients with symptomatic cholelithiasis.

Methodology

After obtaining approval from the hospital’s ethical committee, the Gastrointestinal Quality of Life Index (GIQLI) proforma was filled on admission (T0) and at week six (T1) postoperatively. All data were collected, and GIQLI scores were calculated for individual patients.

Results

In our study, among the 70 patients undergoing LC, 20% (n = 14) were aged 18-30 years and 80% (n = 56) were aged 31-60 years, with the mean ± standard deviation calculated as 41.56 ± 10.13 years. Overall, 44.29% (n = 31) of patients were men and 55.71% (n = 39) were women. GIQLI scores were 94.64 ± 2.24 for pre-treatment and 106.09 ± 2.40 for post-treatment, with a mean change of 11.44 ± 3.29, and a p-value of 0.001, showing a significant difference.

Conclusions

The mean improvement in QoL after LC in patients with symptomatic cholelithiasis is significantly higher when compared with pretreatment.

## Introduction

Gallstone disease (cholelithiasis) is a wide spectrum of conditions, ranging from asymptomatic cholelithiasis, biliary colic, empyema gallbladder, and gangrene to perforation and peritonitis [1]. In other words, it can be categorized as lithogenic state, asymptomatic gallstones, symptomatic gallstones, and complicated gallstones [2]. Cholelithiasis affects 5-22% of the Western population [3]. The Asian and African populations show a lower prevalence. In Pakistan, 10.2% of the population has gallstones [4]. In the United States, 6.5% of males and 10.5% of females have gallstones [1]. This gender difference is attributable to estrogen, which increases biliary cholesterol secretion [2]. The incidence of gallstone formation increases with age. Symptoms occur in only 10-30% of the patients [3], and 1-4% of patients per year are at risk of developing complications [1].

Cholecystectomy is one of the most common surgical procedures performed worldwide, with >750,000 cases in the United States reported annually [5]. With the advent of laparoscopic surgery, approximately 90% of elective and 70% of emergency cholecystectomies are performed laparoscopically [1]. Since the first laparoscopic cholecystectomy (LC) performed by Mouret in France in 1987 [6], it has gained acceptance as the gold standard for the management of uncomplicated symptomatic cholelithiasis [3]. It has now become the second most common general surgical procedure post-appendectomy [7]. In Pakistan, such a procedure was first performed in 1991 by Dr. Mumtaz Mehar. LC offers benefits over the open procedure in terms of reduced postoperative pain, reduced analgesic requirement, better cosmesis, shorter hospital stay, and earlier recovery, with fewer postoperative complications and mortality [1,5,6,8]. LC has been proven to be a safe procedure with a mortality rate of 0.22-0.4% [9].

Health-related quality of life (HRQoL), a rather unknown aspect two decades ago, is now a vital component of medical research [10]. Despite its acknowledged worth, a conceptual definition of this term is lacking [11] Broadly, it entails the physical, emotional, and social functioning status of the human body. Postoperative recovery and quality of life (QoL) are essential components that predict a patient’s hospital stay, return to physical strength, emotional status, and routine activities, as well as define the financial burden on the patient and healthcare facility [12]. The core purpose is to determine the patient’s satisfaction level pre and postoperatively and, repeatedly thereafter, accurately assess the effectiveness of an intervention in terms of long-term well-being. Hence, patient-reported outcomes, such as pain and QoL, are essential considerations from a surgeon’s perspective in opting for a surgical procedure [12]. QoL assessment allows further research and modification of a specific surgical procedure [13].

For as commonly performed surgery as LC, little evidence has been reported on postoperative changes in QoL [7,12]. Although not standardized, the Gastrointestinal Quality of Life Index (GIQLI) is a widely used QoL measuring index for biliary tract diseases, comparing baseline and interval postoperative values to assess the improvement in QoL post-LC. Mertens et al. and Carraro et al. concluded that LC improves gastrointestinal symptoms and QoL in symptomatic cholelithiasis using the GIQLI index [3,8]. Other published studies provide conflicting data and rate cholecystectomy as an overused procedure, suggesting that the postoperative course may be altered by confounding events not sufficiently controlled or recognized [8]. Moreover, overall improvement in postoperative QoL is attributable to the preoperative functional status of the patient [14]. The QoL score was 96 ± 20.2 pretreatment and 108 ± 16.8 post-LC in one study [15].

This study aimed at evaluating changes in QoL post-LC using GIQLI to justify its use as the standard procedure for the treatment of symptomatic gallstone disease. Preoperative GIQLI scores were compared with postoperative scores to determine whether LC improves the QoL. Moreover, this study also aimed to determine the mean improvement in QoL post-LC in patients with symptomatic cholelithiasis.

## Materials and methods

This study was conducted in the General Surgical Unit (hepatobiliary, colorectal, breast, endocrine, and vascular surgery) of a tertiary care center in Pakistan from July 17, 2015, to January 16, 2016.

Using the Epi Info calculator and significance of 0.05, power of 80%, a confidence level of 95%, preoperative mean of 96 ± 20.2, and postoperative mean of 108.6 ± 16.8 (reference statistics) [15], the minimum required sample size was calculated to be 70. Sampling was done using non-probability consecutive sampling.

Inclusion and exclusion criteria

Patients of both genders aged 18-60 years were included in the study. All included patients had sonological evidence of cholelithiasis (ultrasonography showing echoes) with symptoms (any/all of the following: nausea, vomiting, flatulence, dyspepsia, and/or biliary colic). Patients undergoing uncomplicated LC and who consented to participate in the study were included.

The following patients were excluded from the study: those with complicated cholelithiasis/acute diseases (cholecystitis, pancreatitis, gangrene, perforation, or peritonitis); those who converted to open cholecystectomy or complicated LC (bile duct injury, vascular injury, Mirizzi syndrome, or malignancy); those with choledocholithiasis (intraoperative cholangiogram or common bile duct exploration); those with major comorbidity and/or American Society of Anesthesiologists grade of >3 (uncontrolled diabetes mellitus, hypertension, myocardial infarction, respiratory or renal failure, stroke, or chronic liver disease); those who underwent emergency LC; those with gallstones of >3 cm in size on ultrasound scan; those with contraindications to laparoscopy; those with previous upper abdominal surgery; pregnant patients; and those with psychiatric disorders. All these criteria could act as confounding variables and introduce bias.

After obtaining approval from the hospital’s ethical committee (Shaheed Zulfiqar Ali Bhutto Medical University, 28-07-2014), the study and its objectives were explained to consecutive patients admitted to the ward for LC who met the inclusion criteria. Then, patients were interviewed after obtaining consent for study participation. GIQLI proforma along with patient profile and contact number was filled on admission (T0). After a detailed history and physical examination, a baseline preoperative workup was performed, including an electrocardiogram and a chest X-ray in selected patients. Anesthesia fitness was obtained preoperatively, and patients were listed for surgery. Preoperatively, patients underwent a standard four-port LC. Another proforma was filled out at week six (T1) postoperatively by the doctor in charge during the patient’s follow-up visit. All data were collected, and GIQLI scores were calculated for each patient.

Statistical analysis

The Statistical Package for the Social Sciences version 17 (SPSS Inc., Chicago, IL, USA) was used for data analysis. The frequency and percentages for qualitative variables (e.g., gender) and the mean and standard deviation (SD) for quantitative variables (e.g., age and GIQLI scores) at baseline and six weeks and changes were calculated pre and postoperatively. A paired sample t-test was applied between pre and post-mean GIQLI changes. Effect modifiers such as age and gender were controlled through stratification. Post-stratification paired sample test was applied. P-values of <0.05 were considered statistically significant.

## Results

A total of 70 patients fulfilling the inclusion/exclusion criteria were enrolled to determine the mean improvement in QoL post-LC in patients with symptomatic cholelithiasis. Overall, 20% (n = 14) of patients were aged between 18 and 30 years, whereas 80% (n = 56) were aged between 31 and 60 years, with the mean ± SD calculated as 41.56 + 10.13 years (Table 1).

**Table 1:** Age distribution of study patients. SD: standard deviation

Age (in years)	Number of patients	%
18–30	14	20
31–60	56	80
Total	70	100
Mean ± SD	41.56 ± 10.13	

Of the 70 patients, 44.29% (n = 31) were males and 55.71% (n = 39) were females (Table 2).

**Table 2:** Gender distribution of the study patients.

Gender	Number of patients	%
Male	31	44.29
Female	39	55.71
Total	70	100

The GIQLI scores of patients were calculated as 94.64 ± 2.24 for pretreatment and 106.09 ± 2.40 for post-treatment, with the mean change calculated as 11.44 ± 3.29 (p = 0.001), showing a significant difference (Table 3).

**Table 3:** GIQLI scores of the patients. GIQLI: Gastrointestinal Quality of Life Index; SD: standard deviation

GIQLI	Mean	SD
Pre-treatment	94.64	2.24
Post-treatment	106.09	2.40
Mean change	11.44	3.29

Stratification for age and gender was calculated and is presented in Table 4 and Table 5, respectively.

**Table 4:** Stratification for GIQLI scores of the patients with regards to age (n = 70). GIQLI: Gastrointestinal Quality of Life Index; SD: standard deviation

GIQLI	Mean	SD
Pre-treatment	94.64	2.53
Post-treatment	105.79	2.42
GIQLI	Mean	SD
Pre-treatment	94.64	2.20
Post-treatment	106.16	2.42

**Table 5:** Stratification for GIQLI of the patients with regards to gender (n = 70). GIQLI: Gastrointestinal Quality of Life Index; SD: standard deviation

GIQLI	Mean	SD
Pre-treatment	94.26	2.41
Post-treatment	106.06	2.29
GIQLI	Mean	SD
Pre-treatment	94.95	2.10
Post-treatment	106.10	2.52

## Discussion

Cholecystectomy is one of the most common procedures performed worldwide and is becoming increasingly well known in developed countries. Based on the prevalence of gallbladder disease, the moderate variation in the numbers of cholecystectomies performed in various countries cannot be explained. Increasing emphasis is being placed on measuring patient-reported outcomes (including HRQoL) for determining the success of any medical or surgical intervention for any disease. The number of studies performed to measure HRQoL in gastrointestinal medicine and surgery to establish the appropriateness of any intervention is increasing.

This study aimed to evaluate QoL changes post-LC using GIQLI to justify its use as the standard procedure for the treatment of symptomatic gallstone diseases. Preoperative GIQLI scores were compared with postoperative scores to determine whether LC improves the QoL.

In our study, among the 70 patients undergoing LC, 20% (n = 14) were aged between 18 and 30 years, whereas 80% (n = 56) were aged between 31 and 60 years, with a mean ± SD of 41.56 ± 10.13 years. Overall, 44.29% (n = 31) of the patients were males and 55.71% (n = 39) were females. GIQLI scores were 94.64 ± 2.24 for pretreatment and 106.09 ± 2.40 for post-treatment, and the mean change was calculated as 11.44 ± 3.29 (p = 0.001), showing a significant difference.

The study findings are in agreement with a previous study reporting that the QoL score was 96 ± 20.2 pretreatment and 108 ± 16.8 post-LC in one study [15].

Mosimann [16] examined HRQoL using both GIQLI and Short Form Health Survey (SF-36). Patients were divided into groups depending upon the diagnosis (complicated symptomatic cholelithiasis, uncomplicated symptomatic and asymptomatic cholelithiasis) and surgical risk categories. A questionnaire was filled out before and three months post-cholecystectomy. Cholecystectomy was found to be the effective treatment modality in symptomatic gallstones and low surgical risk patients (high QoL gains). HRQoL did not show massive improvement in asymptomatic and high-risk patients. On the other hand, Kitano et al. [17] reported notable improvements in GIQLI scores in both symptomatic and asymptomatic gallstone groups. However, symptomatic patients showed marked improvements in QoL, suggesting patients with lower preoperative GIQLI scores benefit the most from LC. Thus, LC has been shown to be the appropriate treatment for symptomatic and low-risk patients.

Alternatively, Cuschieri examined acute cholecystitis versus symptomatic uncomplicated gallstone disease [18]. The study compared the outcomes between conservative management and surgery using QoL and pain surveys. Patients filled in questionnaires at baseline (preoperatively) and at six, 12, and 60 months postoperatively. The group with no intervention showed a higher rate (36% vs. 19%) of gallstone-related complications; however, the difference was not significant. After randomization, no significant differences were found in pain or QoL measurements. It was shown that QoL outcomes and pain measurements were not significantly affected by conservative management. Thus, a non-operative treatment strategy would be an option for high-risk patients.

Another study from Taiwan reported that symptomatic patients scored lower on SF-36 preoperatively; LC significantly improved the GIQLI scores [19].

However, some studies suggest that certain digestive issues persisted even postoperatively. Indeed, only a few studies showed a drop in SF-36 at 12 months postoperatively; thus, they found different QoL evaluators postoperatively. Two such markers are preoperative direct bilirubin level and drain in Morison’s pouch. This finding supports the fact that poorer preoperative conditions result in maximum GIQLI and QoL gains; moreover, variables that may act as confounding events should be identified [20]. Postoperative outcomes might be affected by other variables (bloating, indigestion, etc.) that were not taken into account and could label cholecystectomy as a glorified treatment option.

However, findings based on other studies reveal that QoL post-LC using GIQLI justifies its use as the standard procedure for the treatment of symptomatic gallstone disease. Further studies are required to validate our findings.

Our study had a few limitations. First, the sample size was not enough to generalize the findings. Second, the confounding factors were not adequately identified and controlled. Moreover, other conditions causing similar symptoms were not taken into account.

## Conclusions

The mean improvement in QoL after LC in patients with symptomatic cholelithiasis is significantly higher when compared with pretreatment. Further large-scale studies need to be conducted to better objectify the usefulness of LC as a treatment strategy for cholelithiasis.

## Appendices

**Table 6:** GIQLI questionnaire. GIQLI: Gastrointestinal Quality of Life Index

Variable	All of the time	Most of the time	Some of the time	A little of the time	Never
1. How often during the past 2 weeks have you had pain in the abdomen?					
2. How often during the past 2 weeks have you had a feeling of fullness in the upper abdomen?					
3. How often during the past 2 weeks have you had bloating (sensation of too much gas in the abdomen)?					
4. How often during the past 2 weeks have you been troubled by excessive passage of gas through the anus?					
5. How often during the past 2 weeks have you been troubled by strong burping or belching?					
6. How often during the past 2 weeks have you been troubled by gurgling noises from the abdomen?					
7. How often during the past 2 weeks have you been troubled by frequent bowel movements?					
8. How often during the past 2 weeks have you found eating to be a pleasure?					
9. Because of your illness, to what extent have you restricted the kinds of food you eat?					
10. During the past 2 weeks how much have you been troubled by the medical treatment of your illness?					
11. How often during the past 2 weeks have you been troubled by fluid or food coming up into your mouth (regurgitation)?					
12. How often during the past 2 weeks have you felt uncomfortable because of your slow speed of eating?					
13. How often during the past 2 weeks have you had trouble swallowing your food?					
14. How often during the past 2 weeks have you been troubled by urgent bowel movements?					
15. How often during the past 2 weeks have you been troubled by diarrhea?					
16. How often during the past 2 weeks have you been troubled by constipation?					
17. How often during the past 2 weeks have you been troubled by nausea?					
18. How often during the past 2 weeks have you been troubled by blood in the stool?					
19. How often during the past 2 weeks have you been troubled by heartburn?					
20. How often during the past 2 weeks have you been troubled by uncontrolled stools?					

Options

All of the time: ≤24 hours a day

Most of the time: ≤18 hours a day

Some of the time: ≤12 hours a day

A little of the time: ≤8 hours a day

Never: 0 hours a day

Score calculation

Most desirable option: 4 points

Least desirable option: 0 points

GIQLI score

Maximum score: 80 points

Minimum score: 0 points
